# Effects of a 1-Year Physical Activity Intervention on Markers of Hemostasis among Breast Cancer Survivors: A Randomized Controlled Trial

**DOI:** 10.1055/s-0040-1721782

**Published:** 2021-02-06

**Authors:** Siv Kjølsrud Bøhn, Inger Thune, Vidar Gordon Flote, Hanne Frydenberg, Gro Falkenér Bertheussen, Anders Husøy, Frøydis Fjeldheim, Sonja Hjellegjerde Brunvoll, Anette Hjartåker, Marie-Christine Mowinckel, Per Morten Sandset, Per Ole Iversen

**Affiliations:** 1 Faculty of Chemistry, Biotechnology and Food Sciences, Norwegian University of Life Sciences, Ås, Norway; 2 Department of Oncology, Oslo University Hospital, Oslo, Norway; 3 Institute of Clinical Medicine, Faculty of Health Sciences, University of Tromsø, Tromsø, Norway; 4 Department of Physical Medicine and Rehabilitation, St. Olav University Hospital, Trondheim, Norway; 5 Department of Neuromedicine and Movement Science, Norwegian University of Science and Technology, Trondheim, Norway; 6 Department of Sports Medicine, Norwegian School of Sport Sciences, Oslo, Norway; 7 Department of Nutrition, Institute of Basic Medical Sciences, University of Oslo, Oslo, Norway; 8 Department of Haematology, Oslo University Hospital and University of Oslo, Oslo, Norway

**Keywords:** Breast Neoplasms, hemostasis, physical activity, postmenopause, von Willebrand factor

## Abstract

**Introduction** Physical activity may reduce the development of breast cancer. Whereas hypercoagulability has been linked to adverse outcomes in breast cancer patients, the effects of physical activity on their hemostatic factors are unknown. The study aimed to assess whether long-term (1 year) physical activity can affect hemostatic factors in breast cancer patients.

**Methods** Fifty-five women (35–75 years) with invasive breast cancer stage I/II were randomized to a physical activity intervention ( *n* = 29) lasting 1 year or to a control group ( *n* = 26), and analyzed as intention to treat. Fibrinogen, factor VII antigen, tissue factor pathway inhibitor, and von Willebrand factor (VWF) antigen as well as prothrombin fragment 1 + 2, the endogenous thrombin potential and D-dimer, were measured in plasma before intervention (baseline), and then after 6 and 12 months.

**Results** Maximal oxygen uptake (measure of cardiorespiratory fitness) decreased the first 6 months among the controls, but remained stable in the intervention group. We found no significant differences between the two study groups regarding any of the hemostatic factors, except a significantly higher increase in factor VII antigen in the intervention group. The effect of the intervention on VWF was, however, significantly affected by menopausal stage, and a significant effect of the intervention was found on VWF among postmenopausal women, even after adjustment for dietary intake.

**Conclusion** Long-term physical activity had no effect on the majority of the hemostatic factors measured, but led to increased plasma concentrations of factor VII antigen and prevented an increase in VWF concentration after breast cancer treatment in postmenopausal women. The clinical impact of these findings for risk of vascular thrombosis warrants further studies.

## Introduction

Breast cancer ranks as the most common malignancy in the adult female population. 1 Due to the steady increase in breast cancer incidence combined with successful treatment, these patients constitute the largest cancer survivor group in general. Important risk factors for breast cancer are age, genetic susceptibility, reproductive therapy, hormonal treatment use as well as overweight/obesity, and high intake of alcohol. The risk of breast cancer doubles each decade up until menopause where the increase tapers off. Breast cancer is, however, more common after menopause compared with premenopause. 2 According to recent data from the World Cancer Research Fund, there is strong evidence that being physically active decreases the risk of breast cancer in both pre- and postmenopausal women, 3 but is not known whether increased physical activity modifies the course of the disease after diagnosis. The mechanisms of how physical activity may reduce breast cancer development and possibly disease progression, are yet not fully understood and key factors include estrogen, energy balance, insulin resistance and -sensitivity, chronic low inflammation as well as DNA repair processes. 4

Cancer is associated with a prothrombotic state with subsequent increased risk of thromboembolic events. 5 Venous thrombosis (VT) is recognized as a leading cause of cancer-associated mortality with a four to seven times higher prevalence in cancer patients compared with the general population. 6 Although the risk of VT is lower in breast cancer compared with some other cancers, this serious complication also affects breast cancer patients, 7 8 and the association between malignancy and the development of hypercoagulability in these patients has been recognized for more than a century. 5 In addition, the breast cancer treatment itself—including surgery, radiation, chemotherapy, and biotherapy—may also render the patients prothrombotic, thus leading to increased risk of VT. Whereas inactivity is a well-known risk factor for VT, and conversely, increased physical activity may counteract this risk 9 ; it is unclear to what extent physical activity can decrease the risk of VT in breast cancer patients. While vigorous activity may cause acute increases in thrombotic factors, 10 regular physical activity is associated with lower risk of cardiovascular disease and associated thrombotic events. 11 12 13 Thus, it has been suggested that at least some of the beneficial effects of regular physical activity is exerted through effects on the hemostatic system. 12 13

Increasing physical activity as a remedy to decrease the levels of coagulation factors may thus provide a possible strategy to restore the hemostatic imbalance in breast cancer patients, which in turn may have implications on disease progression. However, the knowledge of how physical activity affects blood coagulation is incomplete, 14 and no randomized clinical trial has been conducted to explore its effects on the hemostatic balance among breast cancer patients. In this randomized trial, we therefore investigated whether physical activity could affect markers of the hemostatic system the first year after a breast cancer diagnosis.

## Methods

### Participants, Study Design, and Randomization

Between 2011 and 2013, women aged 35 to 75 years diagnosed with ductal carcinoma in situ grade 3 or invasive breast cancer stages I to II were recruited to the parallel-randomized controlled trial before surgery, and randomized 10 ± 2 days after surgery. The current study included patients treated at Oslo University Hospital or at Vestre Viken Hospital in Drammen. The inclusion required adequate Norwegian language skills and the ability to complete the 12-month physical activity intervention. We excluded patients with (1) severe heart disease; dysregulated diabetes mellitus; thyroid disorders; and muscular, skeletal, or other disorders preventing them from perform regular physical activity, (2) a body mass index (BMI) >40 kg/m ^2^ , (3) previous bariatric surgery, and (4) a travel distance >1.5 hours from their home to the intervention site (for practical/logistical reasons).

After completion of the baseline assessments, the patients were randomly allocated 1:1 to either the intervention or to the control group. A central coordinator ( https://www.ntnu.no/mh/akf/randomisering ) performed the randomization. The design and nature of the study did not allow for blinding to group allocation, for neither the study clinicians nor the patients. The research coworkers performing the laboratory analysis and statistics were, however, blinded to group allocation until the data analysis was performed.

The current study was a feasibility trial related to the project entitled “energy balance and breast cancer aspects-II” (EBBA-II) that was approved by the Regional Committee for Medical and Health Research Ethics in Norway (2011/500a). All patients gave informed written consent. EBBA-II is registered with ClinicalTrials.gov, identifier NCT02240836. The current study was analyzed as per intention to treat and is reported according to the CONSORT recommendations ( Supplementary Material ).

### Patient Treatment

All patients were included, and assessments were performed prior to any treatment at the outpatient clinic. Thereafter, they underwent surgical removal of the tumor prior to start of the exercise intervention. The excised tumors were characterized histologically and immunohistochemically and classified according to TNM, histological type, grade, and receptor status as described. 15 The subsequent treatment was given according to the current existing national guidelines developed by the Norwegian Breast Cancer Group. 16 Patients undergoing adjuvant chemotherapy received a combination of fluorouracil, epirubicin, and cyclophosphamide (FEC) or epirubicine and cyclophosphamide (EC), every third week for four to six cycles, or four cycles of either FEC or EC, and/or taxanes (every third week/weekly for 12 weeks). Patients treated with breast-conserving surgery and patients with axillary nodal macrometastasis received radiation therapy, and endocrine therapy was started in hormone receptor positive patients if indicated.

### The Physical Activity Intervention

The exercise program was developed particularly for this trial and based on national and international exercise expertise and programs. 17 18 19 It included aerobic endurance training of moderate-to-high intensity as well as stretching/weight-bearing activities. Each patient randomized to the intervention group had an initial individual session with a trained physiotherapist, and received a detailed individualized training program based on their own general clinical and physical function/capacity and their maximal oxygen uptake (VO _2max_ ). 20 21 These participants were then organized into training groups—with 8 to 12 patients in each group—according to physical function and geography, and started exercising in this group 21 to 28 days postsurgery. These training sessions in groups were taking part outdoors for 60 minutes two times per week during the 12-month trial period, and the participants were in addition required to perform at least 120 minutes of exercise at home, resulting in a total of 240 minutes of exercise weekly. The control group could exercise without any restriction, and both groups (intervention and control groups) received standard of care. VO _2max_ was measured directly at baseline before any treatment and then at 6 and 12 months postsurgery, with a cardiopulmonary exercise test using a modified Balke treadmill protocol.

### Dietary Recordings

The dietary intake was monitored by 7-day precoded food diaries in both study groups 2 weeks after surgery and then after 6 and 12 months. We used a modified and validated version of a food diary developed by the University of Oslo. 22 23 To estimate portion sizes, we used a validated photo-booklet together with household units. 24 All foods, drinks, and supplements consumed were self-reported. The completed food diaries were manually reviewed for irregularities and missing values by nutritionists, and whenever necessary the participants were contacted to obtain the missing value. The completed food diaries were scanned by using the Cardiff TeleForm program version 10.5.1 (Datascan Oslo, Norway) and then computerized by using the KBS calculation software system (“Kostberegningsystem”) and AE-14 food database (predominantly based on the Norwegian food composition table from 2014; http://www.norwegianfoodcomp.no ).

### Measurement of Hemostatic Factors

Venous blood samples were collected after an overnight fast and before surgery, and then at 6 and 12 months after surgery, in tubes containing 0.5 mL sodium citrate (0.105 M). Platelet-poor plasma samples obtained after centrifugation were aliquoted and stored at −80°C until further analysis. The Calibrated Automated Thrombogram (CT; Diagnostica Stago, Asnières, France), which measures thrombin generation as a function of time, was measured as previously described. 25 26 To increase reproducibility, all CT-derived parameters were expressed in percentage of the measured test value of a reference plasma pooled from 34 healthy individuals run in the same assay. In our assay, the concentration of tissue factor, 5 pM hemostatic markers, were measured by using commercial ELISA assays: von Willebrand factor (VWF) antigen, total and free tissue factor pathway inhibitor antigen, and D-dimer, (Cat.no. 00942, 00261, 00262, and 00947, respectively, Diagnostica Stago); factor VII antigen (FVIIAg; ELISA kit RK036A, Hyphen BioMed, Neuville-sur-Oise, France), and prothrombin fragment 1 + 2 (F _1+2_ ; ELISA kit OPBD03, Siemens Healthcare Diagnostics Products GmbH, Marburg, Germany). Fibrinogen was measured according to the clotting method of Clauss (kit 00673, Diagnostica Stago) by using the STA-R analysis instrument (Diagnostica Stago).

### Assessment of Clinical Variables

Clinical data were assessed before any treatment except for the assessment of dietary intake, which was performed after surgery. Trained nurses and senior oncologists recorded data on surgery, radiation, chemotherapy, and antihormonal treatment. A modified version of the Norwegian EBBA-I study questionnaire was used to collect general information including menstrual and reproductive history, birth weight, and lifestyle habits. 15 Premenopausal status was defined as having regular menstruation, whereas a postmenopausal status was defined as having no menstruation for the past ≥12 months or having a postmenopausal hormonal status. Patients aged ≥55 years were considered postmenopausal if there was uncertainty about postmenopausal status due to hormone replacement therapy.

### Statistical Analyses

The change in the plasma concentration of D-dimer, a marker of activated coagulation was chosen as the main outcome of the current study. We considered that a 50% reduction in the D-dimer level between the intervention and control groups after 1 year would be relevant. This would correspond to a difference between D-dimer means of 555 and 370 ng/mL. The latter was reported as median value for healthy controls. 27 Hence, with a power of 80% and a significance level of 5%—an estimated SD of 221—calculated from the 75% percentile of the healthy controls, 27 we needed a minimum of 23 patients in each group.

Comparisons of characteristics between the intervention and control group were performed by using the two-sample Student's *t* -test for continuous variables and Pearson's Chi-squared test for categorical variables. When frequencies were below 1 or if less than 80% of the cells had expected frequencies larger than 5, Fisher's exact test was used. Effects of the intervention were tested by using the mixed model with random intercept in IBM SPSS 24 treating time as a categorical parameter, with the following parameterization: β0 time + β1 group + β2 (time × group) We evaluated the main effects of time and group and their interaction on 6 and 12 months data with the baseline levels as covariate. The difference between the two study groups are given in the text where the fixed effect of group was significant. Significance was assumed for *p* <0.05. The interaction (time × group) was removed from the model when insignificantly affecting the model. The three-way interaction (time × group × menopausal stage) was also tested to justify subgroup analysis separately on pre- and postmenopausal women.

## Results

### Characteristics of the Two Study Groups

**Fig. 1:**
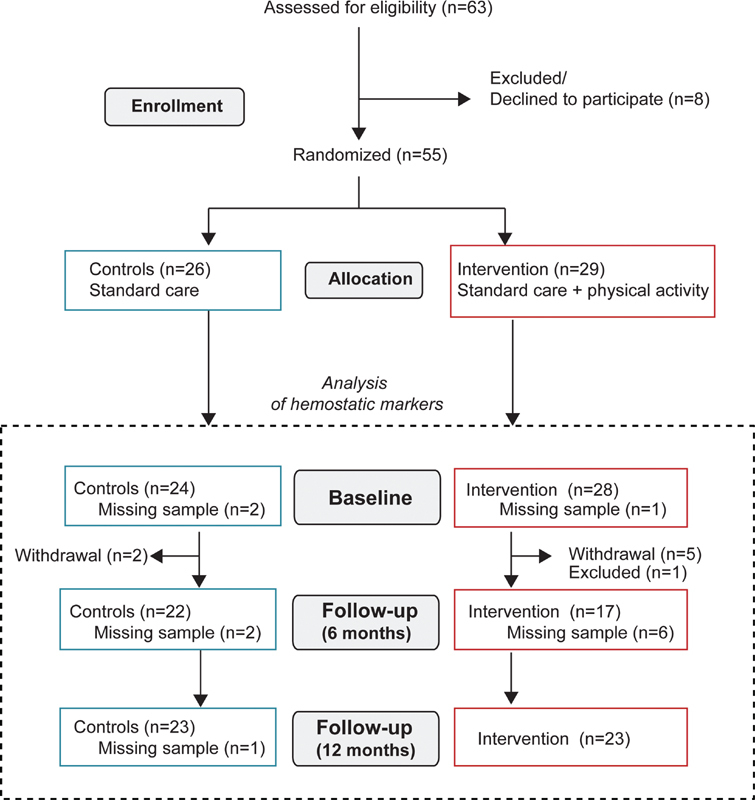
Flow chart showing the inclusion process of the patients in the study. Maximum number of available samples for the analyses of hemostatic markers is indicated. Note that the number of missing samples was higher for some of the hemostatic markers, as indicated in the tables.

After assessing 63 patients for eligibility, eight were excluded according to the enrollment criteria. Thus, 55 patients were randomized to the intervention ( *n* = 29) or control group ( *n* = 26; Fig. 1 ). Two patients in the intervention group and one in the control group withdrew from the study after randomization. The baseline characteristics of the two study groups are presented in Table 1 , whereas tumor characteristics and treatment modalities are presented in Tables 2 and 3 , respectively. Compared with the intervention group, the proportion of patients having children was lower in the control group ( *p* = 0.01) and the age at conceiving the first child was higher ( *p* < 0.01). A higher proportion of patients in the control group received breast-conserving treatment ( *p* = 0.03) compared with the intervention group. However, for most of the characteristics, there were no significant differences between the two study groups, indicating that a balanced study group allocation was achieved. No adverse events were reported during the duration of the study.

**Table 1:** Patient characteristics at baseline

	Intervention	Control	*p* -Value
	** ( *n * = 29) **	** ( *n * = 26) **	
Age (y)	55.7 ± 7.8	54.3 ± 7.7	0.50 a
Body mass index (kg/m ^2^ )	24.8 ± 3.1	25.0 ± 3.6	0.81 a
VO _2_ max (mL/kg/min) (median [range])	30.1 (32.0)	28.9 (35.8)	0.32 b
Daily smoking (%, *n* )	24.1 (7)	23.1 (6)	1.00 d
Education (y)	15.6 ± 3.2	16.8 ± 3.3	0.18 a
Menopausal stage (%, *n* )			
Premenopausal	34.5 (10)	26.9 (7)	0.54 c
Postmenopausal	65.5 (19)	73.1 (19)	
Parity (%, *n* )	82.8 (24)	50.0 (13)	0.01 b
Age at first child birth (median [range])	27 (19)	31(14)	< 0.01 b
Contraceptive use (%, *n* )	86.2 (25)	80.8 (21)	0.72 d
Hormone replacement therapy (%, *n* )	41.4 (12)	30.8 (8)	0.41 d

Abbreviation: VO _2_ max, maximal oxygen uptake.

Note: Values are mean ± standard deviation unless otherwise stated.

a Student's *t* -test.

b Mann–Whitney test.

c Pearson's Chi-squared test.

d Fisher's exact test ( *n* = 24/13).

**Table 2:** Tumor characteristics

	Intervention	Control	*p* -Value
	( *n * = 29)	( *n * = 26)	
Side (%, *n* )			
Right	48.3 (14)	65.4 (17)	0.17 b
Left	51.7 (15)	30.8 (8)	
Both	0 (0)	3.8 (1)	
Histology (%, *n* )			
NST/ductal	79.3 (23)	80.8 (21)	0.56 b
Lobular	13.8 (4)	7.7 (2)	
DCIS	6.9 (2)	3.8 (1)	
Other	0 (0)	7.7 (2)	
Grade (%, *n* )			
I	25.9 (7)	20 (5)	0.83 a
II	51.9 (14)	52 (13)	
III	22.2 (6)	28 (7)	
Tumor stage (%, *n* )			
DCIS, grade III	6.9 (2)	3.8 (1)	0.90 b
T1, 1–20 mm	65.5 (19)	73.1 (19)	
T2, 21–50 mm	27.5 (8)	23.1 (6)	
Tumor diameter (mm)	17 (32)	12 (36)	0.12 c
Node stage (%, *n* )			
N0 (0)	67.9 (19)	80.8 (21)	0.63 b
N1 (1–3 nodes)	25 (7)	19.2 (5)	
N2 (4–9 nodes)	3.6 (1)	0 (0)	
N3 (>10 nodes)	3.6 (1)	0 (0)	
Estrogen positive (%, *n* )			
Yes	89.7 (26)	84.6 (22)	0.49 b
Progesterone positive (%, *n* )			
Yes	79.3 (23)	76.9 (20)	0.89 b
HER2 positive (%, *n* )			
Yes	0 (0)	7.7 (2)	0.40 b
Ki67% hot spot index	19 (80)	21 (76)	0.48 c

Note: Data are presented as median (range) or *n* (%).

Abbreviations: DCIS, ductal carcinoma in situ; NST, invasive carcinoma of no special type.

a Pearson's Chi-squared test.

b Fisher's exact test.

c Mann–Whitney test.

**Table 3:** Treatment for breast cancer among the patients

	Intervention	Control	*p* -Value
	( *n * = 29)	( *n * = 26)	
Surgery (%, *n* )			
Breast-conserving treatment	58.6 (17)	84.6 (22)	0.03 a
Mastectomy	41.4 (12)	15.4 (4)	0.03 a
Sentinel node examination	72.4 (21)	80.8 (21)	0.47 a
Axillary dissection	27.6 (8)	19.2 (5)	0.47 a
Radiation therapy (%, *n* )			
Breast			
40/50 Gy	69 (20)	84.6 (22)	0.17 a
Lymph nodes			
40/46 Gy	17.2 (5)	15.4 (4)	1.00 b
Chemotherapy (%, *n* )	55.2 (16)	65.4 (17)	0.44 a
Antracycline treatment	55.2 (16)	65.4 (17)	0.44 a
Taxanes + FEC	34.5 (10)	46.2 (12)	0.38 a
Endocrine treatment	75.9 (22)	57.7 (15)	0.15 a
Tamoxifen treatment	34.5 (10)	26.9 (7)	0.55 a
AI treatment	41.4 (12)	30.8 (8)	0.42 a
Traztuzumab treatment	0 (0)	7.7 (2)	0.22 b

Abbreviations: AI, aromatase inhibitor; FEC, fluorouracil, epirubicine and cyclophosphamide.

Note: One case of mastectomy with primary reconstruction.

a Pearson's Chi-squared test.

b Fisher's exact test.

**Fig. 2:**
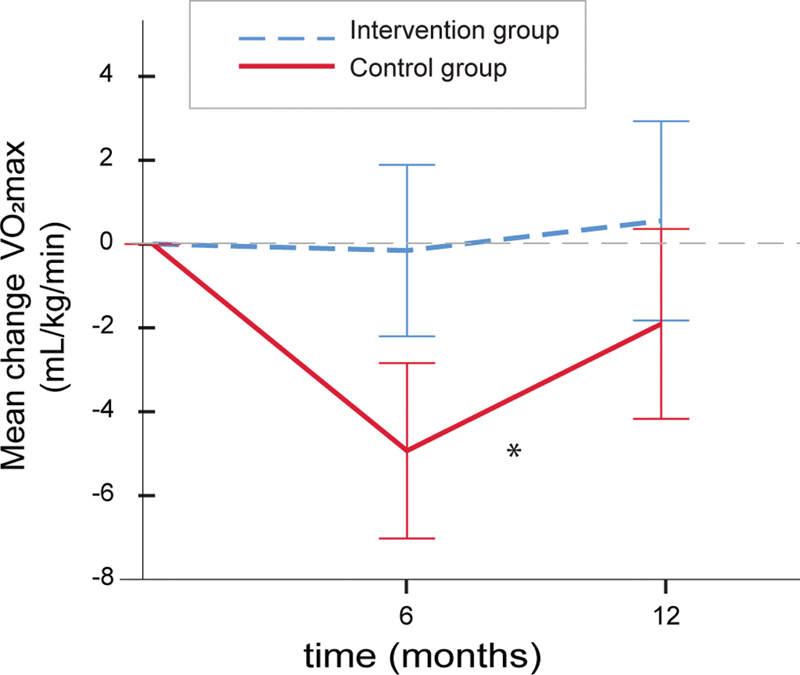
Maximal oxygen uptake in the two study groups during the study period. The lines show mean (95% confidence intervals) differences from baseline in VO _2max_ levels in the intervention group (blue stippled line, *n* = 29/22/21 at 6 and 12 months, respectively) and the control group (red line, *n* = 25/24/23 at 6 and 12 months, respectively). The asterisk denotes a significant difference (mixed model adjusting for baseline values,* *p* = 0.005) between the two study groups with regard to the main group effect.

The change in VO _2max_ was significantly different between the groups ( Fig. 2 ) (mixed model analysis, main group effect, *p* = 0.005). The model was not adjusted for or categorized by treatment. Whereas patients in the control group experienced a decrease in VO _2max_ the first 6 months after surgery before it returned toward baseline levels, VO _2max_ among patients in the intervention group remained fairly stable throughout the study period, indicating acceptable compliance to the physical activity intervention.

### Temporal Profiles of the Hemostatic Markers

Samples for hemostatic analysis were available for 24 patients in the control group and 28 participants in the intervention group ( Fig. 1 ). No significant differences were found for the change in hemostatic factors over time between the two study groups except for FVIIAg, which increased more in the intervention group compared with the controls ( Table 4 ). To account for possible dietary changes, a mixed model analysis on FVIIAg was performed with stepwise adjustment for the intake of selected food groups: potatoes (g/day), vegetables (g/day), fruit and berries (g/day), meat (g/day), and fiber (g/day) and total energy intake (J/day). Only intake of fiber and meat affected the model significantly, but the group effects remained significant and *p* -values became even stronger ( *p* = 0.028 and *p* = 0.013 respectively) (data not shown). The mixed model analysis of the data from the CT assay revealed no significant differences between the two study groups ( Table 5 ).

**Table 4:** Hemostatic markers in the two study groups during the period

	Time ( *n* ^int^ / *n* ^ctr^ )	Intervention	Controls	*p-* Value
F _1+2_ (pmol/L)	Baseline (22/21)	210.80 ± 185.96	196.69 ± 60.30	0.11
	6 mo (17/19)	190.88 ± 55.41	235.86 ± 84.04	
	12 mo (22/21)	203.14 ± 57.60	256.19 ± 222.25	
D-dimer (ng/mL)	Baseline (22/21)	349.38 ± 147.48	355.62 ± 140.54	0.26
	6 mo (18/20)	449.34 ± 263.07	458.10 ± 251.50	
	12 mo (22/21)	479.24 ± 311.65	356.87 ± 126.94	
Fibrinogen (g/L)	Baseline (22/21)	3.38 ± 0.62	3.13 ± 0.52	0.70
	6 mo (17/19)	3.36 ± 0.69	3.36 ± 0.56	
	12 mo (21/21)	3.43 ± 0.83	3.12 ± 0.54	
FVIIAg (%)	Baseline (21/21)	100.45 ± 16.75	100.99 ± 14.65	0.046
	6 mo (16/20)	108.65 ± 21.69	103.54 ± 17.23	
	12 mo (21/21)	110.35 ± 20.21	104.82 ± 14.98	
Total TFPI (ng/mL)	Baseline (21/21)	63.47 ± 11.94	61.94 ± 14.49	0.28
	6 mo (16/20)	66.59 ± 15.07	63.16 ± 14.54	
	12 mo (21/20)	65.40 ± 17.05	62.46 ± 14.08	
Free TFPI (ng/mL)	Baseline (21/21)	8.36 ± 2.75	7.93 ± 2.64	0.71
	6 mo (16/18)	10.31 ± 3.45	10.24 ± 3.09	
	12 mo (21/20)	10.72 ± 4.65	9.94 ± 3.55	
vWF (pg/mL)	Baseline (21/21)	114.73 ± 21.80	105.45 ± 18.67	0.26
	6 mo (16/19)	129.24 ± 26.85	126.75 ± 23.79	
	12 mo (21/20)	125.75 ± 26.68	118.08 ± 18.36	

Note: Values are means ± standard deviation. The *p* -values are obtained from the fixed group effect from the ANCOVA mixed model with baseline values as covariates. FVIIAg is measured as % of normal plasma pool. “Time ( *n* ^int^ / *n* ^ctr^ )” represents the time of the sampling and the number of samples available for biomarker analysis from the intervention ( *n* ^int^ ) and control group ( *n* ^ctr^ ).

Abbreviations: F _1+2_ , prothrombin fragment 1 + 2; FVIIAg, coagulation factor VII antigen; TFPI, tissue factor pathway inhibitor; VWF, von Willebrand factor.

**Table 5:** Markers of thrombin generation in the two study groups during the study period

	Time ( *n* ^int^ / *n* ^ctr^ )		Intervention	Controls	*p* -mixed
	Baseline	(28/24)	107.94 ± 18.50	108.82 ± 17.77	0.90
ETP (%)	6 mo	(17/22)	97.41 ± 11.89	100.54 ± 21.74	
	12 mo	(23/23)	101.48 ± 13.74	103.81 ± 18.43	
	Baseline	(28/24)	100.02 ± 25.22	99.03 ± 19.04	0.21
Lag time (%)	6 mo	(17/22)	109.73 ± 26.42	108.09 ± 33.27	
	12 mo	(23/23)	107.07 ± 29.13	97.21 ± 15.98	
	Baseline	(28/24)	118.17 ± 32.97	117.77 ± 24.48	0.37
Peak (%)	6 mo	(17/22)	99.82 ± 18.79	105.42 ± 31.05	
	12 mo	(23/23)	100.12 ± 19.42	108.15 ± 25.97	
	Baseline	(28/24)	99.57 ± 21.49	96.51 ± 15.26	0.73
Time-to-peak (%)	6 mo	(17/22)	106.43 ± 14.33	109.41 ± 39.61	
	12 mo	(23/23)	107.51 ± 21.14	100.74 ± 14.45	
	Baseline	(28/24)	99.27 ± 9.54	99.15 ± 6.78	0.74
Start-tail (%)	6 mo	(17/22)	101.2 ± 5.94	104.15 ± 19.51	
	12 mo	(23/23)	103.68 ± 7.93	99.72 ± 8.88	

Note: Values are % of normal plasma pool and expressed as means ± standard deviation. The *p* -values are obtained from the fixed group effect from the ANCOVA mixed model that adjusts for baseline values as covariates. The markers of thrombin generation are as follows: ETP (endogen thrombin potential), the area under the thrombin generation curve; lag-time, the time interval between the start of the assay and the onset of thrombin generation; peak, the maximum concentration of thrombin; time-to-peak, the time required to reach maximum thrombin concentration and Start-Tail, the time interval of the end of the thrombin generation. “Time ( *n* ^int^ / *n* ^ctr^ )” represents the time of the sampling and the number of samples available for biomarker analysis from the intervention ( *n* ^int^ ) and control group ( *n* ^ctr^ ).

### Menopausal Effects on the Hemostatic Markers

**Fig. 3:**
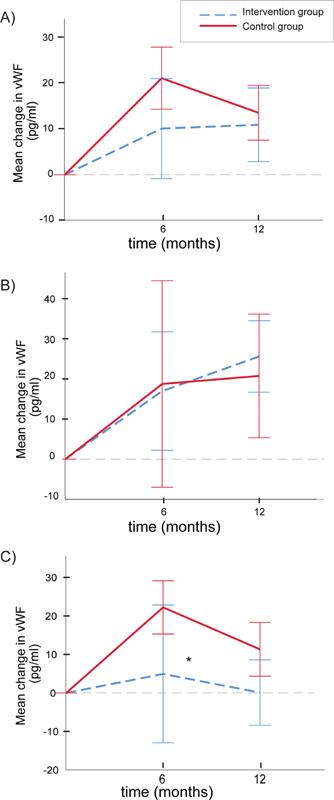
Concentrations of von Willebrand factor in the two study groups during the 1-year observation period. The lines show mean (95% confidence intervals) differences from baseline in the intervention group (blue stippled lines) and the control group (red lines) at 6 and 12 months. Data are shown for the total patient group ( **A** ), the premenopausal ( **B** ), and the postmenopausal women ( **C** ). The asterisk denotes a significant difference between the two study groups with regard to main group effect (mixed model adjusting for baseline values, * *p* = 0.026). The ratios of the number of pre- and postmenopausal women in the control group at time points 0, 6 and 12 months were 5/16, 5/14, and 5/15, respectively. The corresponding ratios of pre- and postmenopausal women in the intervention group were 9/12, 7/9, and 9/15.

Changes in the hemostatic system are associated with the onset of menopause. 28 We therefore tested for a three-way interaction between group, time, and menopausal stage ( Fig. 3A ). Interestingly, a significant interaction was found for VWF ( *p* = 0.001) and a separate analysis was performed in the pre- and postmenopausal women. Notably, whereas no differences in VWF levels ( Fig. 3B ) were observed among premenopausal women; among the postmenopausal women, a significant difference was found between the groups where the controls had a significantly higher increase from baseline compared with the intervention group ( *p* = 0.026 for the marginal fixed group effects, Fig. 3C ).

To account for possible dietary changes, a mixed model analysis on VWF was performed for the postmenopausal women with stepwise adjustment for intake of selected food groups: potatoes (g/day), vegetables (g/day), fruit and berries (g/day), meat (g/day), and fiber (g/day) and total energy intake (J/day). None of the food group intakes nor total energy intake (J/day) affected the model significantly (data not shown).

## Discussion

To the best of our knowledge, this is the first time the effect of physical activity has been studied on markers of hemostasis in a randomized, controlled trial in newly diagnosed breast cancer patients before and after adjuvant treatment. We found that a 12-month intervention with physical activity revealed no significant effect on the levels of D-dimer or other pro-coagulant factors in patients with newly diagnosed invasive breast cancer stage I/II except for FVIIAg, which increased more in the intervention group. We also found a significant effect of menopausal stage on the effect of the intervention on VWF. In the postmenopausal women, the levels of VWF increased more among the controls than in the intervention group while this effect was not observed among the premenopausal women.

To the best of our knowledge, no clinical trials have reported on effects of physical activity on coagulation factors in breast cancer populations (PubMed, search date October 16, 2020 combining the search terms [coagulation OR haemostasis] and [breast cancer] with filter for [clinical trial]). Also, little information seems to be available on the effects of exercise training on blood coagulability as such, particularly effects of long-term exercise and reviews on the topic calls for further investigations. 29 However, the lack of significant effects of long-term physical activity on the majority of the measured hemostatic factors measured in our study stands in contrast to some other studies performed in different study populations. 30 31 32 Besides different study populations, there are also other possible explanations for the discrepancies including variations in type and intensity of the physical activity programs employed and the use of different analytical methods to measure hemostatic factors.

The finding that the concentrations of FVIIAg in plasma increased more in the intervention group compared with the control group was somewhat unexpected, as this factor is instrumental in the coagulation cascade, has been associated with cardiovascular events, and is also associated with aspects of cancer progression. 33 FVII circulates in the blood stream in two molecular forms: single-chain zymogen FVII (FVIIz) and the double-chain activated FVII (FVIIa). While we have measured the total plasma concentration of the protein (FVIIAg), which includes both molecular forms, it is only FVIIa that activates the coagulation cascade. Some cross-sectional studies show that habitual physical activity is associated with lower plasma FVIIa, but the results from intervention studies are inconsistent. In contrast with our findings, Gris et al found significant reductions in FVIIa after a 3-month physical training program designed to reduce weight. 34 Thus, the weight loss or dietary changes may have been responsible for the effects on FVIIa. Other studies are in line with our results. Wosornu et al found a gradual rise in FVIIa after aerobic training while finding a small fall in the group exposed to power exercise training after coronary artery surgery. 35 Zanettini et al examining effects of physical activity in a small group of sedentary subjects found no effects on FVII, although beneficial impacts were found for their coronary risk profile. 36

An interpretation of the current results on FVIIAg changes could be that physical activity leads to adverse hemostatic effects via blood FVII concentrations. However, the highest FVIIAg levels (mean % of normal ± SD) in the intervention group found at 12 months (110.35 ± 20.21) is still within reference range of Oslo University Hospital. Also, because we have measured the total plasma concentration of FVIIAg and not the activity, the higher FVIIAg does not necessarily indicate a higher coagulation tendency. It might be the activation potential and not the concentration that is more important. Interestingly, the ratio of FVIIa/FVIIAg can predict cardiovascular events and is higher among women in menopause compared with premenopausal women. 37 Notably, the impact of FVII on coagulation status among breast cancer patients is still unclear. Only one clinical trial was found on PubMed (search date September 27, 2020) combining the search terms (FVII) and (breast cancer) and (clinical trial). 38 This trial examined the effect of very low-dose warfarin on markers of hypercoagulation in metastatic breast cancer and found that FVIIz antigen and, to a lesser extent, FVII total antigen were lowered by warfarin treatment.

The association between VWF levels and long-term effects of physical activity is novel. In addition to being a well-known independent predictor of atherothrombotic disease, 39 VWF has also been linked to various types of cancer and increased levels are associated with poor prognosis. 40 In line with this, a recent observational study in colorectal patients found that the lower levels of VWF were associated with improved event-free survival and less thrombotic complications. 41 Notably, the plasma concentration of VWF is increased among patients with malignant breast disease. 42

In accordance with our findings, a large British prospective study of cardiovascular disease in 3,810 elderly men found that physical activity was significantly and inversely related with VWF in a dose–response manner. 43 In contrast to our study, the authors also found similar effects of physical activity on other hemostatic factors such as fibrinogen, platelet count, coagulation factors VIII and IX, D-dimer, and tissue plasminogen activator antigen, even after adjustment for possible confounders. It is possible that this difference may be explained by a more profound effect of physical activity on hemostatic parameters in men than women. The physical activity measures were also self-reported by using a physical activity (exercise) score while we have used an objective measure (VO _2max_ ). In support of this, VWF concentrations have been found to be significantly higher in men than in women aged 41 to 50 years. 44 Interestingly, no difference was found between men and women in the other age groups, and the authors suggested that this gender-effect in the 41 to 50 age group could be due to the transition into menopause.

Long-term (12 months) effects of physical activity on vascular factors in breast cancer populations have apparently not been previously reported. However, results from a recent 12-months exercise training intervention study confirmed our findings with no effects on markers of hypercoagulability (VWF not included) in patients with combined type 2 diabetes mellitus and coronary artery disease. 45

Why the effect on VWF was only found in postmenopausal and not in the premenopausal women, is unclear, but may be related to a more prothrombotic condition among postmenopausal women in general, and thus, a larger potential for change in VWF. In support of this, an increase in markers of coagulation activation with aging has been reported. 46 Notably, a study of blood donors from South-Wales found that women had a higher rate of increase of VWF in middle age, which could be related to change in hormonal status and passage into menopause. 44 Another explanation for the lack of an effect on VWF in the premenopausal patients may also be related to tamoxifen treatment, which has been reported to favor procoagulation and impair anticoagulation. 47 Because more premenopausal patients were treated with tamoxifen compared with postmenopausal women (data not shown), the effects of physical activity on VWF in the premenopausal women may not be sufficient to counteract the procoagulative effects of the chemotherapy treatment. It is important to consider, however, that the number of premenopausal participants was small.

The main strengths of the present study include the robust trial design with adequate randomization leading to well-balanced study groups, assessment of baseline data before any treatment, solid data on adherence to the physical activity intervention (data of maximal oxygen uptake) as well as detailed monitoring of dietary intakes. The control group had a lower proportion having children, but whether this will impact hemostasis is unknown. In addition, a higher proportion of patients received breast-conserving treatment in the control group, possibly indicating a higher surgical burden in the intervention group which may mask potential effects of the intervention.

In conclusion, we found that a 12-month physical activity intervention significantly increased FVIIAg levels and inhibited an increase in VWF among postmenopausal breast cancer patients. Whether the findings results from a beneficial effect of the physical activity intervention needs to be tested with clinically relevant endpoints, such as recurrence/relapse of the underlying malignancy and/or the presence of thrombosis.
